# Using an introduced index to assess the association between food diversity and metabolic syndrome and its components in Chinese adults

**DOI:** 10.1186/s12872-018-0926-x

**Published:** 2018-10-03

**Authors:** Wenzhi Zhao, Jian Zhang, Ai Zhao, Meichen Wang, Wei Wu, Shengjie Tan, Mofan Guo, Yumei Zhang

**Affiliations:** Department of Nutrition and Food Hygiene, School of Public Health, Peking University Health Science Center, Xueyuan Road 38, Haidian District, 100191 Beijing, China

**Keywords:** Metabolic syndrome, Diet, Healthy food diversity, Adult

## Abstract

**Background:**

It is reported that an increase in food diversity would lower the risk of cardiac–cerebral vascular diseases.

**Methods:**

A new index was introduced to develop a Chinese healthy food diversity (HFD) index, exploring the association with metabolic syndrome (MetS) and its components among Chinese adults. Two sets of data were used. The primary data were from a cross-sectional survey conducted in 2016 called the Chinese Urban Adults Diet and Health Study (CUADHS); the verification data were from the China Health and Nutrition Survey (CHNS) of 2009. The Chinese HFD index was developed according to the Chinese Dietary Guideline, with food consumption information from 24-h dietary recalls. The association between the index and MetS and its components was explored in logistic regression models.

**Results:**

Among 1520 participants in the CUADHS, the crude prevalence of MetS was 36.4%, which was 29.0% after the standardisation of age and gender by the 2010 Chinese national census. In the CUADHS, the HFD index ranged from 0.04 to 0.63. The value of the index among participants who are male, young, poorly educated, drinking or smoking, and with high energy intakes was significantly lower than that of their counterparts. In the verification dataset of the CHNS, there were 2398 participants, and the distribution of different genders and age groups was more balanced. The crude prevalence of MetS in the CHNS was 27.3% and the standardised prevalence was 19.5%. The Chinese HFD index ranged from 0.02 to 0.62. In the CUADHS, the Chinese HFD index was not significantly associated with MetS in covariate-adjusted models or with its components. In the CHNS, the Chinese HFD index had a significantly negative correlation with MetS and its components (i.e., elevated fasting glucose and elevated waist circumference) in covariate-adjusted models.

**Conclusions:**

Increased food diversity may decrease the risk of MetS, which is important in dietary interventions of cardiac–cerebral vascular disease. This underscores the necessity of continued investigation into the role of HFD in the prevention of MetS and provides an integral framework for ongoing research.

**Electronic supplementary material:**

The online version of this article (10.1186/s12872-018-0926-x) contains supplementary material, which is available to authorized users.

## Background

Metabolic syndrome (MetS) is a clustering of risk factors for cardiovascular disease (CVD) and type 2 diabetes and has been a public health challenge globally [1–3]. People diagnosed with MetS have a five-time higher risk of developing type 2 diabetes and are twice as likely to develop CVD within the next 5–10 years [4, 5]. It has been reported that the occurrence of MetS increases total mortality by 1.5 times and cardiovascular death by 2.5 times [5, 6]. The prevalence of MetS is striking in both developed and developing countries. The National Health and Nutrition Examination Survey (NHANES) of the United States showed that the prevalence of MetS among adults increased from 25.3% in 1988–1994 to 34.2% in 2007–2012. The Korean National Health and Nutrition Examination Survey (KNHANES) from 2008 to 2013 found a stable prevalence of 28.9%. In China’s most recent national survey in 2009, a prevalence of 21.3% was reported [6–8]. It should be noticed that the prevalence of MetS in different areas of China was largely altered, ranging from 20 to 45% [9–11].

Environmental factors, including diet, exercise, stress, and certain addictions (tobacco or alcohol), as well as genetic factors are crucial in the development of MetS [12–14]. Lifestyle modification, such as dietary intervention, is recommended to manage MetS. Studies have reported that some dietary components influence MetS directly, as either protective factors or risk factors [15–19]. There are many researches focusing on the association between dietary pattern or kinds of indices evaluating dietary quality and MetS. However, it is difficult for most people to develop and sustain healthful dietary patterns with individual knowledge and self-control, particularly given that transformations in diet environments have expanded access to all kinds of food [12, 20–22]. The increasing dietary variety comes with benefits and challenges. On the one hand, it avoids malnutrition; on the other hand, it may result in overweight and obesity [23–25].

Dietary guidelines recommended by government institutions or national nutrition associations provide guidance to the public and serve as referential files when designing dietary and nutritional interventions. Researchers in Germany and the US developed and validated a healthy food diversity (HFD) index based on actual food guidelines and explored the correlation between HFD and body adiposity and MetS [26–28]. The US researchers found that the HFD index values were inversely associated with indicators of body adiposity; the odds of obesity and android-to-gynoid ratio > 1 are lower among those with a higher US HFD index [26]. In a randomised-controlled clinical weight-loss trial, participants with a higher US HFD index had greater weight loss and waist circumference (WC) reduction [29]. Besides, the US HFD index was inversely associated with components of MetS, including elevated WC and low HDL cholesterol [27].

The latest version of the dietary guidelines in China was released in 2016, and up to now there is no study introducing the Chinese HFD index. It is necessary and meaningful to develop and evaluate an index considering food type, quality, and consumption amounts simultaneously, and to explore its association with health conditions in Chinese adults in order to guide dietary interventions.

In this study, we developed the Chinese HFD index based on the Dietary Guidelines for Chinese Residents and then examined its associations with MetS and its components in urban adults.

## Methods

### Study design and participants

There were two sets of data in the analysis. The primary data were from a cross-sectional survey designed and conducted by our team, the Chinese Urban Adults Diet and Health Study (CUADHS), conducted from March to July 2016, in which a multi-stage sampling method was utilised to recruit adult subjects. Firstly, eight cities were selected based on geographical location and economic status, including two first-tier cities with higher economic performance. Secondly, two communities from each first-tier city and one community from each non-first-tier city were chosen by convenience sampling. In the last step, subjects were recruited by age groups, with at least 60 people from the age group of 18–44 years, 60 from 45 to 64 years, and 50 from over 65 years in each community. The study recruited 1806 subjects, and those with a physical disability, mental illness, or memory problems and women who were pregnant were excluded from analysis. In the end, a total of 1520 subjects were considered eligible for this study. We obtained a formal written agreement from each participant.

The verification data were from the China Health and Nutrition Survey (CHNS) of 2009. The CHNS is an international collaborative project between the National Institute for Nutrition and Food Safety of the Chinese Centre for Disease Control and Prevention, and the University of North Carolina at Chapel Hill. As a longitudinal, household-based survey, it was conducted in 1989, 1991, 1993, 1997, 2000, 2004, 2006, 2009, and 2011 in sequence. Only the survey conducted in 2009 contained information about blood biochemical examination. Details are provided elsewhere [30]. Questionnaires and blood biochemical tests were used to collect information of adults (except pregnant women) living in urban areas (i.e., residence in city and town or county capital city). In this study, we analysed 2398 individuals from the CHNS who met our inclusion criteria described in the data collection section.

### Data collection

In the CUADHS, the interviewer-administered questionnaires contained questions about socio-demographic characteristics, lifestyles, disease history, health literacy, dietary intakes, and physical examination. Anthropometry measurement and blood tests were also conducted. The food frequency questionnaire (FFQ) and 24-h dietary recall for 1 day were used in this study to collect dietary intake information. The FFQ was a semi-quantitative questionnaire, in which 41 groups of food were asked about regarding the frequency and amount of consumption within the past month. Intakes of different kinds of alcohol and beverages were included. One-time 24-h dietary recall was used to obtain the data on food intakes, and then nutrient intakes were calculated based on it. About 1/8 (205 adults) of the subjects were invited to complete the 24-h dietary recall for 3 days to evaluate the representativeness of one-time 24-h dietary recall. Energy and nutrient intakes were calculated and analysed based on the Chinese Food Composition Tables (CFCT) of 2004 and 2009 (CFCT, National Institute of Nutrition and Food Safety, China CDC), the Standard Tables of Food Composition in Japan (2010), and the nutrient composition table on the food packaging. Training of the interviewers and a pilot investigation were completed prior to data collection.

Questionnaire information and blood biochemical indices of the CHNS 2009 were downloaded from the official website of the CHNS, and information was gathered about socio-demographic characteristics, lifestyles, disease history, 24-h dietary recalls for 3 days, consumption of household food inventory, energy and macronutrient intakes, anthropometry information, and blood biochemical indices labelled by specimen collection and processing by the China–Japan Friendship Hospital (CJFH).

### Assessment of diet factors and development of the Chinese HFD index

For the CUADHS, we calculated energy and nutrient intakes based on CFCT, Standard Tables of Food Composition in Japan [31], and ingredient lists of common supplements. Then we computed the absorbed amount of special micronutrients by bioavailability adjustment, the detailed processing procedure of which is described elsewhere [32]. We referred to the FAO protocol [33] to calculate the probabilities of nutrient adequacy (PA) of some nutrients based on Chinese Dietary Reference Intakes (DRIs) 2013 edition, which included definitions and specified values for estimated average requirements (EAR) and recommended nutrient intakes (RNI). The equation was PA = Probnorm [(estimated intakes-EAR)/CV] for most nutrients except iron; the CV was set to 10% of EAR for all nutrients except vitamin A (20%), niacin (15%), and zinc (25%).

For iron, since its distribution was not normal, we used the eq. PA = estimated participant’s intake/RNI. PA was defined as the ratio of a certain nutrient intake to its recommended allowance, and when intakes of a certain nutrient exceeded requirement, the value should be capped at 1, indicating 100% adequacy. We calculated the PA of protein, carbohydrates, vitamin A, niacin, vitamin B6, vitamin B1, vitamin B2, vitamin B12, vitamin C, folate, calcium, iron, and zinc. We then calculated the mean probability of adequacy (MPA) of micronutrients by applying equal weight to every individual micronutrient.

We evaluated the representativeness of one-time 24-h dietary recall in the CUADHS, and the results are showed in Additional file 1: Table S1. There were no significant differences in energy and macronutrient intakes between one-time 24-h dietary recall and three-day 24-h dietary recall. We decided that one-time 24-h dietary recall was able to represent the results of 24-h dietary recall for 3 days, which was similar to other studies [34–36].

In the CHNS the food intakes of every participant were the summation of two parts. Firstly, daily average food intake was calculated from three-day 24-h dietary recalls. Secondly, for food consumed at home, the daily amount of each ingredient from the household food inventory consumed by each individual was estimated based on his/her respective proportion of energy intake in the whole family.

For the two studies, all of the food items were divided into 15 groups according to the Dietary Guideline and Balance Diet Pagoda for Chinese Residents (as showed in Table 1), and, subsequently, the consumption of each food group was calculated, respectively. Though there were only 12 food groups in the Balance Diet Pagoda, in the detailed Dietary Guidelines there were precise recommended daily/weekly intakes of 15 food groups, in which cereals, tubers, and beans were divided into three groups: whole grains and legumes, tubers, and refined grains. Soya and nuts were divided into two groups: soya products and nuts and seeds [37]. According to similar research [28, 38], weight based on the recommended proportions of each food group at the 2000-kcal level in the Dietary Guideline and Balance Diet Pagoda for Chinese Residents was selected to build the Chinese HFD index (Table 1). There was no precise definition for dark green and yellow or orange vegetables in the recommendation, so we redefined them as vegetables rich in vitamin A (content of vitamin A ≥ 150 RAE/100 g; RAE = retinol equivalent) or vitamin C (content of vitamin C ≥ 50 mg/100 g). The Chinese HFD index values were calculated using the following algorithm:

**Table 1:** Development of health factors (hf) for each food group according to recommendations based on the energy intake of 2000 kcal

Food groups	Recommended amount (g)	Share of food group (broad or single)	hf
Emphases	1300	0.81	
Whole grains and legumes	100	0.08	0.06
Tubers	75	0.06	0.05
Other vegetables	225	0.17	0.14
Vitamin A- or vitamin C-rich vegetables^a^	225	0.17	0.14
Fruits	300	0.23	0.19
Dairy^b^	300	0.23	0.19
Soya products^c^	15	0.01	0.01
Nuts and seeds	10	0.01	0.01
Aquatic products	50	0.04	0.03
Includes	275	0.17	
Refined grains	150	0.55	0.09
Meat and poultry	50	0.18	0.03
Eggs	50	0.18	0.03
Oil	25	0.09	0.02
Limits	31	0.02	
Salt	6	0.19	0.00
Added sugar	25	0.81	0.02

Note: ^a^ = dark green and yellow or orange vegetables in the Dietary Guideline and Balance Diet Pagoda for Chinese Residents;

^b^= liquid milk based on food exchanging of all kinds of dairy

^c^= soybean based on food exchanging of all kinds of soy foods

Chinese HFD index=$\left(1-\sum {S}_i^2\right)\times \mathrm{hv}$,

in which $$ {S}_i=\frac{\  Amount\ of\ recommended\ food\ group\kern0.5em i\  by\  weight\ }{\  Amount\ of\ 15\  food\ groups\  by\  weight} $$,

hv =  ∑ *hf*_*i*_ × *S*_*i*_, *hf*_*i*_ = *Broad food share* × *share of food group*,

where *S*_*i*_ is the quantitative share of a single food group; hv is the health value; and *hf*_*i*_ is health factors.

Theoretically, the range of the Chinese HFD index is between 0 (i.e., a diet with a single food group) and nearly 1 (i.e., a more balanced diet). In fact, the maximum hv that can be achieved according to the recommendation at the 2000-kcal level is 0.187, and the Chinese HFD index was calibrated by dividing hv by its maximum. Then, the Chinese HFD index values and energy intakes were equally divided into four groups (Q1, Q2, Q3, and Q4).

### Assessment of non-dietary factors

We categorised age groups as 18~ 44.9 years old, 45~ 54.9 years old, 55~ 64.9 years old, and ≥ 65 years old. Smoking status was classified as never smoked, former smoker, and current smoker. Drinking behaviours were defined as not drinking and drinking now. Physical activity (PA) levels calculated from a PA questionnaire were trisected from the smallest to the highest and marked as T1, T2, and T3, which were expressed as metabolic equivalents (MET)/d; subjects who only reported sedentary behaviours were classified as a separate group. Educational background was classified as illiterate, middle school or lower, high school or professional training, undergraduate, and postgraduate student or higher. Body mass index (BMI) was defined as weight (in kilograms) divided by the squared height (in metres). Consistent with the WHO criteria, obesity, overweight, normal, and underweight were defined as BMI ≥28 kg/m^2^, BMI ≥24 kg/m^2^ and < 28 kg/m^2^, BMI ≥18.5 kg/m^2^ and < 24 kg/m2, and BMI<18.5 kg/m^2^, respectively [39].

### Diagnostic criteria of MetS

The definition of MetS was consistent with the most recent Joint Interim Statement (JIS) by adopting the Asian criteria for WC recommended by the International Diabetes Federation. Indicators of MetS are shown in Table 2, and subjects were diagnosed as patients if they had at least three of the indicators [5]. The methods of measuring anthropometry indicators and blood biochemical indices are presented in Additional file 2: Table S2.

**Table 2:** Criteria for clinical diagnosis of metabolic syndrome

No.	Measure	Categorical Cut Points
1	Elevated waist circumference (WC)	≥90 cm in males; ≥85 cm in females
2	Elevated triglycerides (TC) (drug treatment for elevated triglycerides is an alternate indicator)	≥150 mg/dL (1.7 mmol/L)
3	Reduced high-density lipoprotein cholesterol (HDL-C) (drug treatment for reduced HDL-C is an alternate indicator)	<40 mg/dL (1.0 mmol/L) in males;<50 mg/dL (1.3 mmol/L) in females
4	Elevated blood pressure (antihypertensive drug treatment in a patient with a history of hypertension is an alternate indicator)	Systolic ≥130 and/or diastolic ≥85 mmHg
5	Elevated fasting glucose (drug treatment of elevated glucose is an alternate indicator)	≥100 mg/dL

### Statistical analysis

Normality was examined before the related analysis. Values were presented as mean ± standard deviation ($\overline{x}\pm SD$) for continuous variables or as number (percentage) for categorical variables. Student t tests or one-way ANOVA were performed to compare means between different groups. Chi-square tests or trend Chi-square tests were performed to compare the distribution of categorical variables. Kendall’s tau-b correlation was tested between PA/MPA and the Chinese HFD index, with energy intake as a covariate. Binominal unconditional logistic regression models were used to estimate the effects of factors on MetS and the regression coefficients (ORs), and their 95% confidence interval (CI) was obtained. All statistical analyses were performed using the Statistic Package for Social Science (SPSS) version 20.0 (SPSS Inc., Chicago, IL, USA). *P* values lower than 0.05 were considered statistically significant.

## Results

### Demographic characteristics and the prevalence of MetS and its components

There were 1520 adults analysed in the CUADHS, of which 527 were male. Those who were 18~ 44.9 years old and older than 65 years accounted for about 1/3 of the total, respectively. The educational attainment of about 70% participants was high school or lower. About half of the participants never smoked and were non-drinking now. The prevalence of overweight and obesity was 35.4% and 11.0%, respectively. The detailed distribution of demographic characteristics is showed in Table 3.

**Table 3:** General characteristics of participants and the distribution of MetS and its components of CUADHS

Factor	Group	N	Elevated WC		Elevated fasting glucose		Elevated blood pressure		Elevated TC	Reduced HDL			MetS	
			%	P	%	P	%	P	%	P	%	P	%	P
Total		1520,100	35.3		32.9		37		40.9		43.6		36.4	
Gender	Male	527,34.7	41.2	<0.001	41.7	<0.001	46.1	<0.001	47.2	<0.001	42.5	0.548	43.5	<0.001
	Female	993,65.3	32.1		28.2		32.2		37.5		44.1		32.7	
Age	18~ 44.9 years	547,36	17.6	<0.001	13.7	<0.001	9.7	<0.001	20.8	<0.001	30.2	<0.001	14.6	<0.001
	45~ 54.9 years	260,17.1	36.2		24.6		26.9		40		43.5		29.2	
	55~ 64.9 years	272,17.9	48.2		46		55.5		56.6		56.3		52.6	
	≥65 years	441,29.0	48.8		53.5		65.5		56.5		52.4		57.8	
Educational attainment	Illiterate	66,4.3	63.6	<0.001	47	<0.001	69.7	<0.001	62.1	<0.001	60.6	<0.001	69.7	<0.001
	Middle school or lower	445,29.3	46.5		45.4		51.9		49.4		47.4		46.3	
	High school or professional training education	627,41.3	32.7		28.4		32.4		40.4		46.1		34	
	Undergraduate education or higher	378,24.9	21.2		23		21.2		28.3		32.3		23	
Drinking behavior	No	942,68.5	36.3	0.427	34.9	0.594	41.3	0.027	44.8	0.188	45.8	0.315	39.7	0.212
	Yes	434,31.5	34.1		36.4		35		41		42.9		36.2	
Smoking behavior	Never smoked	1145,75.3	32.3	<0.001	30.2	<0.001	34.4	<0.001	38.4	0.002	42.4	0.066	33.1	<0.001
	Former smoker	165,10.9	46.1		46.7		53.3		44.8		42.4		46.1	
	Current smoker	210,13.8	42.9		36.7		38.6		51		51		47.1	
BMI	<18.5 kg/m2	74,4.9	0	<0.001	9.5	<0.001	5.4	<0.001	4.1	<ss0.001	12.2	<0.001	4.1	<0.001
	18.5~ 24.0 kg/m2	739,48.6	7		26		26.9		29.8		33		18.3	
	24.0~ 28.0 kg/m2	538,35.4	60.8		41.4		48.1		53.9		56.1		53.9	
	≥28.0 kg/m2	168,11.1	92.9		46.4		60.1		64.3		63.7		75	
Physical activity	Sedentary group	119,7.8	38.7	0.095	33.6	0.008	32.8	0.001	45.4	0.132	50.4	0.169	40.3	0.092
	T1	468,30.8	30.8		28.6		31		36.5		41		31.8	
	T2	466,30.7	36.3		31.1		38		42.7		45.9		38.6	
	T3	467,30.7	37.9		38.8		43.3		42.2		42		37.9	
Energy intake	Q1	380,25.0	35.8	0.964	30.3	0.121	35.3	0.647	37.6	0.485	41.3	0.71	33.7	0.639
	Q2	380,25.0	35.8		35.5		35.8		41.8		44.5		37.1	
	Q3	380,25.0	34.2		29.7		37.9		42.9		45.3		37.4	
	Q4	380,25.0	35.3		36.1		39.2		41.1		43.2		37.6	
HFD-index	Q1	368,24.2	33.2	0.764	29.9	0.166	36.1	0.295	38.6	0.196	37.5	0.014	35.3	0.035
	Q2	410,27.0	36.6		36.8		39		41.2		46.1		37.1	
	Q3	368,24.2	35.1		27.4		33.4		38.3		41.8		31.5	
	Q4	374,24.6	36.1		36.9		39.3		45.2		48.4		41.7	

Note: ^a^Subjects who only reported sedentary behaviors were classified as Sedentary group, the others were trisected into three groups (T1, T2 and T3) according to the calculated metabolic equivalents (MET)/d from the smallest to the highest;

^b^Energy intakes were quadrisected into four groups (Q1, Q2, Q3 and Q4) from the smallest to the highest;

^c^Chinese HFD-Index values were quadrisected into four groups (Q1, Q2, Q3 and Q4) from the smallest to the highest

Table 3 also shows that in the CUADHS the prevalence of MetS was 36.4%, which was 29.0% after standardisation of age and gender by the 2010 Chinese national census. Significant differences in the prevalence of MetS were found between different groups of gender, age, education, smoking behaviour, BMI, and HFD index. The prevalence of MetS and its five components was significantly higher in participants who are male, older, poorly educated, a former or current smoker, and overweight or obese.

In the verification data of the CHNS, 2398 participants were analysed, and the distribution of gender (47.9% male) and age groups (30.9%, 25.4%, 20.7%, and 23.0%, respectively) was more balanced than that of the CUADHS. For the educational attainment, more than 85% of the participants were high school or lower. About 70% of the participants never smoked and were non-drinking now. The prevalence of overweight and obesity was 32.7% and 11.1%, respectively. Detailed information is shown in Table 4.

**Table 4:** General characteristics of participants and the distribution of MetS and its components of CHNS

Factor	Group	N	Elevated WC		Elevated fasting glucose		Elevated blood pressure		Elevated TC		Reduced HDL		MetS	
			%	P	%	P	%	P	%	P	%	P	%	P
Total		2398	36.2		33.1		33.3		36.2		28.1		27.3	
Gender	Male	1106	37	0.485	35.5	0.018	36.7	<0.001	42	<0.001	21.3	<0.001	28.7	0.171
	Female	1292	35.6		31		30.3		31.2		33.9		26.2	
Age	18~ 44.9 years	742	22.8	<0.001	20.1	<0.001	10	<0.001	32.1	0.014	28	0.935	14.3	<0.001
	45~ 54.9 years	608	33.7		31.7		27.1		36.3		27.6		23.8	
	55~ 64.9 years	496	45.4		40.7		42.5		40.9		29.2		35.9	
	≥65 years	552	48.9		45.1		63		37.3		27.7		40.9	
Educational attainment	Illiterate	192	52.1	<0.001	42.7	<0.001	61.5	<0.001	32.8	0.311	29.2	0.929	40.6	<0.001
	Middle school or lower	1145	40.1		37.7		36		37.8		28.6		30.9	
	High school or professional training education	755	28.1		26		27.3		34.3		27.7		20.4	
	Undergraduate education or higher	303	32.3		27.1		20.1		37.3		27.1		22.8	
Drinking behavior	No	1646	36.6	0.614	33.4	0.661	33.8	0.441	34	<0.001	30.9	<0.001	27.7	0.527
	Yes	752	35.5		32.4		32.2		41		21.9		26.5	
Smoking behavior	Never smoked	1735	36.7	0.788	32	0.226	31.9	<0.001	33.8	<0.001	30.5	<0.001	26.5	0.332
	Former smoker	83	34.9		36.1		55.4		38.6		19.3		31.3	
	Current smoker	580	35.2		35.7		34.1		42.9		22.1		29.1	
BMI	<18.5 kg/m2	116	2.6	<0.001	17.2	<0.001	15.5	<0.001	9.5	<0.001	17.2	<0.001	2.6	<0.001
	18.5~ 24.0 kg/m2	1221	14.2		26.9		25.6		28.3		23.8		14.9	
	24.0~ 28.0 kg/m2	779	56.6		38.5		41.1		45.7		30.8		37.7	
	≥28.0 kg/m2	264	92.8		53		52.7		55.7		44.7		64.8	
Physical activity^a^	Sedentary group	1132	43.3	<0.001	38.3	<0.001	42.8	<0.001	38.1	0.356	29.2	0.225	33.9	<0.001
	T1	463	31.3		29.8		30.9		34.8		27.6		24.4	
	T2	544	28.5		27.6		20.6		34.4		28.7		20.4	
	T3	258	30.6		27.9		22.9		34.5		22.9		18.2	
Energy intake^b^	Q1	691	35.6	0.442	34.6	0.622	37.5	0.023	34.7	0.152	30	0.093	28.7	0.752
	Q2	678	37.5		32		33.3		35.3		28.6		26.5	
	Q3	580	34		33.8		29.8		40.2		29		27.6	
	Q4	449	38.3		31.4		31.2		34.7		23.4		26.1	
HFD-index^c^	Q1	479	40.3	0.013	36.1	0.141	31.9	0.668	39.2	0.265	25.9	0.275	28.8	0.046
	Q2	519	39.5		35.3		35.1		37.6		27.6		30.3	
	Q3	579	35.1		31.8		34		35.2		31.1		28.3	
	Q4	821	32.6		30.8		32.4		34.2		27.6		23.9	

Note:^a^Subjects who only reported sedentary behaviors were classified as Sedentary group, the others were trisected into three groups (T1, T2 and T3) according to the calculated metabolic equivalents (MET)/d from the smallest to the highest;

^b^Energy intakes were quadrisected into four groups (Q1, Q2, Q3 and Q4) from the smallest to the highest;

^c^Chinese HFD-Index values were quadrisected into four groups (Q1, Q2, Q3 and Q4) from the smallest to the highest

The crude prevalence of MetS in the CHNS was 27.3%, and the standardised prevalence was 19.5%. The prevalence of MetS was significantly higher in participants who are older, poorly educated, overweight or obese, and lack PA, while no similar trend was found in the five components of MetS.

### Distribution of the Chinese HFD index and its correlations with nutrients

In the CUADHS, the Chinese HFD index ranged from 0.04 to 0.63. The values of the index were significantly lower in participants who are male, young, poorly educated, drinking or smoking now, and with high energy intakes (Table 5).

**Table 5:** Distribution of Chinese HFD-index in participants of two studies

Factor	Group	CUADHS		CHNS	
		$\overline{x}\pm s$	P	$\overline{x}\pm s$	P
Total		0.41 ± 0.10		0.38 ± 0.06	
Gender	Male	0.38 ± 0.10	<0.001	0.37 ± 0.06	<0.001
	Female	0.42 ± 0.10		0.38 ± 0.06	
Age	18~ 44.9 years	0.39 ± 0.10	<0.001	0.37 ± 0.06	0.001
	45~ 54.9 years	0.41 ± 0.10		0.38 ± 0.06	
	55~ 64.9 years	0.40 ± 0.10		0.38 ± 0.06	
	≥65 years	0.42 ± 0.10		0.38 ± 0.07	
Educational attainment	Illiterate	0.39 ± 0.09	0.018	0.36 ± 0.06	<0.001
	Middle school or lower	0.40 ± 0.10		0.37 ± 0.06	
	High school or professional training education	0.42 ± 0.10		0.39 ± 0.07	
	Undergraduate education or higher	0.41 ± 0.10		0.41 ± 0.07	
Drinking behavior	Yes	0.40 ± 0.10	0.001	0.37 ± 0.06	<0.001
	No	0.42 ± 0.10		0.38 ± 0.06	
Smoking behavior	Never smoked	0.42 ± 0.10	<0.001	0.38 ± 0.06	<0.001
	Former smoker	0.39 ± 0.11		0.38 ± 0.07	
	Current smoker	0.37 ± 0.09		0.37 ± 0.06	
BMI	<18.5 kg/m2	0.40 ± 0.09	0.367	0.38 ± 0.06	0.875
	18.5~ 24.0 kg/m2	0.41 ± 0.10		0.38 ± 0.06	
	24.0~ 28.0 kg/m2	0.41 ± 0.10		0.38 ± 0.06	
	≥28.0 kg/m2	0.40 ± 0.10		0.37 ± 0.07	
Physical activity^a^	Sedentary group	0.40 ± 0.09	0.443	0.38 ± 0.06	<0.001
	T1	0.41 ± 0.10		0.38 ± 0.06	
	T2	0.41 ± 0.10		0.38 ± 0.06	
	T3	0.41 ± 0.10		0.37 ± 0.06	
Energy intake^b^	Q1	0.41 ± 0.10	<0.001	0.38 ± 0.07	<0.001
	Q2	0.42 ± 0.10		0.38 ± 0.06	
	Q3	0.41 ± 0.09		0.38 ± 0.06	
	Q4	0.39 ± 0.10		0.36 ± 0.06	

Note:^a^Subjects who only reported sedentary behaviors were classified as Sedentary group, the others were trisected into three groups (T1, T2 and T3) according to the calculated metabolic equivalents (MET)/d from the smallest to the highest;

^b^Energy intakes were quadrisected into four groups (Q1, Q2, Q3 and Q4) from the smallest to the highest

To validate the Chinese HFD index, correlations between the Chinese HFD index and PA of nutrients were analysed, and the results are showed in Table 6. The Chinese HFD index was positively correlated with PA of carbohydrates, vitamin B2, niacin, vitamin B6, folate, vitamin A, vitamin C, and MPA of micronutrients after adjusting for energy.

**Table 6:** Correlations between Chinese HFD-index and nutrients

Nutrients		Correlation coefficient	
		Crude	Energy adjusted
Macronutrient	PA(Protein)	−0.027	0.047
	PA(Carbohydrates)	−0.03	0.078**
Micronutrient	PA(VitaminB1)	0.016	0.044
	PA(VitaminB2)	0.288**	0.280**
	PA(Niacin)	−0.032	0.054*
	PA(VitaminB6)	0.172**	0.072**
	PA(VitaminB12)	0.034	0.037
	PA(Folate)	0.179**	0.206**
	PA(VitaminA)	0.293**	0.249**
	PA(VitaminC)	0.381**	0.296**
	PA(Zn)	0.075**	0.015
	PA(Ca)	0.378**	0.018
	PA(Fe)	−0.043	−0.009
	MPA	0.141**	0.271**

Note: *,*P*<0.05;**,*P*<0.01

In the verification study, the Chinese HFD index ranged from 0.02 to 0.62, as showed in Table 5. Participants who were male, young, poorly educated, drinking or smoking now, obese, and with the highest energy intakes had a significantly lower Chinese HFD index.

### Regression analysis of the Chinese HFD index and MetS and its components

As shown in Table 7, in the CUADHS the Chinese HFD index was not significantly associated with MetS in covariate-adjusted models or its components. Only in an unadjusted model was the Chinese HFD index positively correlated with elevated fasting glucose and reduced HDL.

**Table 7:** Odds of MetS and its components across quartiles of the Chinese HFD-index among participants of CUADHS, OR(95%CI)

Item	Q1^3^	Q2^3^	Q3^3^	Q4^3^	P-trend
Elevated WC					
Crude	1	1.163 (0.865,1.564)	1.088 (0.802,1.476)	1.139 (0.841,1.542)	0.486
Model 1^a^	1	1.047 (0.766,1.431)	1.069 (0.773,1.477)	1.002 (0.723,1.387)	0.951
Model 2^b^	1	1.107 (0.796,1.539)	0.996 (0.705,1.405)	0.967 (0.684,1.367)	0.726
Elevated fasting glucose					
Crude	1	1.367 (1.013,1.846)	0.887 (0.644,1.222)	1.371 (1.009,1.863)	0.272
Model 1^a^	1	1.244 (0.898,1.724)	0.878 (0.621,1.243)	1.221 (0.869,1.715)	0.628
Model 2^b^	1	1.270 (0.906,1.780)	0.844 (0.589,1.210)	1.216 (0.855,1.730)	0.726
Elevated blood pressure					
Crude	1	1.131 (0.845,1.513)	0.887 (0.655,1.202)	1.144 (0.85,1.540)	0.728
Model 1^a^	1	0.901 (0.640,1.268)	0.799 (0.559,1.142)	0.84 (0.589,1.198)	0.258
Model 2^b^	1	0.908 (0.636,1.297)	0.764 (0.526,1.110)	0.812 (0.56,1.177)	0.183
Elevated blood pressure					
Crude	1	1.116 (0.837,1.488)	0.989 (0.735,1.330)	1.312 (0.979,1.758)	0.144
Model 1^a^	1	0.992 (0.730,1.349)	0.963 (0.701,1.325)	1.162 (0.845,1.598)	0.427
Model 2^b^	1	0.931 (0.675,1.283)	0.932 (0.669,1.299)	1.127 (0.808,1.573)	0.52
Reduced HDL					
Crude	1	1.425 (1.070,1.899)	1.199 (0.892,1.612)	1.563 (1.166,2.095)	0.012
Model 1^a^	1	1.302 (0.969,1.749)	1.13 (0.832,1.534)	1.356 (0.998,1.843)	0.112
Model 2^b^	1	1.251 (0.914,1.713)	1.096 (0.792,1.517)	1.355 (0.978,1.878)	0.134
MetS					
Crude	1	1.079 (0.805,1.446)	0.843 (0.620,1.145)	1.31 (0.974,1.762)	0.244
Model 1^a^	1	0.913 (0.661,1.259)	0.788 (0.562,1.104)	1.091 (0.783,1.522)	0.848
Model 2^b^	1	0.884 (0.631,1.239)	0.754 (0.529,1.073)	1.074 (0.757,1.523)	0.936

Note: ^a^regression model adjusted age and gender;

^b^regression model adjusted age, gender, drinking behavior, smoking behavior, BMI, physical activity and energy intakes;

^c^Chinese HFD-Index values were quadrisected into four groups (Q1, Q2, Q3 and Q4) from the smallest to the highest

In the verification data of the CHNS, the results were different, as shown in Table 8. The Chinese HFD index was significantly negatively correlated with MetS and its components of elevated fasting glucose and elevated WC in covariate-adjusted models. It indicated that a higher Chinese HFD index decreased the risk of MetS.

**Table 8:** Odds of MetS and its components across quartiles of the Chinese HFD-index among participants of CHNS, OR(95%CI)

Item	Q1^3^	Q2^3^	Q3^3^	Q4^3^	P-trend
Elevated WC					
Crude	1	0.846 (0.672,1.067)	0.710 (0.562,0.899)	0.664 (0.524,0.841)	<0.001
Model 1^a^	1	0.801 (0.631,1.017)	0.650 (0.510,0.829)	0.579 (0.453,0.740)	<0.001
Model 2^b^	1	0.811 (0.638,1.031)	0.671 (0.525,0.858)	0.603 (0.470,0.773)	<0.001
Elevated fasting glucose					
Crude	1	0.893 (0.704,1.132)	0.752 (0.591,0.958)	0.820 (0.645,1.041)	0.077
Model 1^a^	1	0.859 (0.673,1.096)	0.704 (0.549,0.903)	0.744 (0.581,0.954)	0.013
Model 2^b^	1	0.856 (0.671,1.094)	0.701 (0.545,0.901)	0.747 (0.582,0.960)	0.016
Elevated blood pressure					
Crude	1	1.244 (0.98,1.579)	0.965 (0.756,1.232)	1.066 (0.837,1.358)	0.909
Model 1^a^	1	1.214 (0.931,1.584)	0.839 (0.639,1.101)	0.883 (0.674,1.157)	0.101
Model 2^b^	1	1.230 (0.942,1.606)	0.870 (0.661,1.146)	0.908 (0.691,1.194)	0.165
Elevated blood pressure					
Crude	1	0.973 (0.772,1.227)	0.720 (0.568,0.914)	0.837 (0.662,1.059)	0.056
Model 1^a^	1	0.985 (0.779,1.245)	0.733 (0.576,0.933)	0.869 (0.684,1.104)	0.117
Model 2^b^	1	0.977 (0.772,1.236)	0.717 (0.563,0.915)	0.856 (0.672,1.091)	0.096
Reduced HDL					
Crude	1	1.197 (0.927,1.544)	1.190 (0.921,1.537)	1.229 (0.952,1.586)	0.161
Model 1^a^	1	1.157 (0.894,1.497)	1.125 (0.868,1.457)	1.117 (0.862,1.448)	0.53
Model 2^2^	1	1.161 (0.896,1.503)	1.095 (0.844,1.422)	1.094 (0.842,1.422)	0.677
MetS					
Crude	1	1.008 (0.787,1.292)	0.827 (0.641,1.067)	0.791 (0.612,1.022)	0.033
Model 1^a^	1	0.962 (0.744,1.244)	0.758 (0.582,0.987)	0.692 (0.530,0.904)	0.002
Model 2^b^	1	0.964 (0.745,1.247)	0.757 (0.580,0.987)	0.698 (0.533,0.914)	0.003

Note:^a^ regression model adjusted age and gender;

^b^regression model adjusted age, gender, drinking behavior, smoking behavior, BMI, physical activity and energy intakes;

^c^Chinese HFD-Index values were quadrisected into four groups (Q1, Q2, Q3 and Q4) from the smallest to the highest

## Discussion

This study was conducted to analyse the associations between MetS and food diversity in Chinese urban adults, and the analysis was repeated in a representative sample for validation. In the two studies, the prevalence of MetS was within the range of contemporaneous researches. In this study, a multi-dimensional food diversity index in consideration of dietary quality and proportionality was applied to evaluate the associations between food diversity and MetS and its components in Chinese adults. The Chinese HFD index was positively correlated with carbohydrates and micronutrients, meaning that if one had a higher HFD index, he/she was more likely to have adequate nutrient intakes. In comparison, the correlations between the Chinese HFD index and PA of most nutrients were weaker than those of the German population (except vitamin A) and US population (except vitamin C), despite the fact that the two foreign studies neglected bioavailability adjustment of minerals [28, 38]. The Chinese HFD index could reflect dietary quality to some extent.

The mean and range of the Chinese HFD index of participants in the two studies were similar, and the trend by grouping factors was also similar. The Chinese HFD index values in the two studies were comparable with the studies conducted in the US and Germany [27–29, 38]. They were also the same as former studies in which the participants who are old, female, and with higher educational attainment were more likely to have a higher HFD index value [26, 27]. Considering that the CUADHS and the CHNS were two independent surveys, the comparable results indicated the feasibility and applicability of the methods in the Chinese population.

Currently, economic growth and globalisation have increased access to seasonal, animal-source, and processed foods, which may result in the intake of both high- and low-quality foods. Health effects of food variety or diversity are controversial; for example, some researches have showed that greater food variety resulted in increased food consumption and obesity [23, 40, 41], while others have indicated beneficial effects of food variety on weight control [42, 43]. However, the difference in definition of food variety between studies should be noticed. The Chinese HFD index was based on recommended food groups for adults to keep healthy, and it considered three aspects comprehensively: type, amount, and health value of consumed food. Participants with higher Chinese HFD index values showed better adherence to the Dietary Guideline and Balance Diet Pagoda for Chinese Residents, and our assumption was that the higher Chinese HFD index values would favourably influence MetS and its components.

In CUADHS data, we did not find that a higher Chinese HFD index influenced the risk of MetS and its components. In CHNS data, it is significant that the Chinese HFD index was negatively correlated with MetS and its components of elevated fasting glucose and elevated WC, suggesting favourable effects of food diversity on MetS and its components. The Chinese HFD index was negatively correlated with elevated WC, and it was similar with the results of articles exploring the influence of HFD on MetS or obesity [26, 27]. The protective effect of food diversity on weight control was proved in some studies [44–46], and it was supposed that diversified diets would provide adequate vitamins, minerals, and bioactive substances but moderate or restricted energy, leading to more balanced and healthier diets. In our research, we verified that those people with the lowest Chinese HFD index had the highest energy intakes.

There are researches exploring the correlations between food diversity and glucose homeostasis, suggesting that higher food diversity decreased the risk of diabetes or impaired glucose homeostasis [44, 47, 48]. The protection mechanism was partly attributed to its favourable effects on weight control, and further research was needed to explore the mechanism accurately.

Our research showed that the Chinese HFD index was negatively correlated with MetS and some of its components, indicating that the increase in food diversity would decrease the risk of MetS. Although we failed to find correlations between the Chinese HFD index and hypertension or dyslipidemia, results of other researches support the premises that higher food diversity lowers cardiovascular risk and an increase in food diversity is important in dietary interventions against chronic non-communicable diseases [47].

There were some limitations of our analysis that cannot be ignored. Firstly, we failed to incorporate family medical history or genetic factors into the analysis. Secondly, we applied one-day or three-day 24-h dietary recall to represent the general condition of food intakes, and the representativeness was limited and recall bias was unavoidable. Thirdly, in a cross-sectional study we can only prove association rather than causal relationship between the Chinese HFD index and MetS and its components. However, this was the first analysis based on the Dietary Guideline and Balance Diet Pagoda for Chinese Residents regarding HFD and MetS in Chinese urban adults. The Chinese HFD index developed by this study was comparable to that of other countries and made it possible to measure compliance with dietary guidelines quantitatively.

## Conclusions

This study quantitatively described the status of MetS and the Chinese HFD index, and found that the Chinese HFD index was negatively associated with MetS and some of its components, such as elevated WC and elevated fasting glucose. Increased food diversity may lower the risk of MetS, which is important in designing dietary interventions for cardiac–cerebral vascular diseases. The study underscores the necessity of continued investigation into the role of healthful food diversity in the prevention of MetS and provides an integral framework for ongoing research.

## Additional files

Additional file 1: **Table S1.** Comparison of energy and macronutrients calculated from mean value of three-day 24-h dietary recalls and one-time 24-h dietary recall in the CUADHS. (DOCX 14 kb)

Additional file 2: **Table S2.** Measurement methods of anthropometry indicators and blood biochemical indices. (DOCX 15 kb)

## Abbreviations

ANOVA: analysis of variance
BMI: body mass index
CFCT: Chinese Food Composition Tables
CHNS: China Health and Nutrition Survey
CI: confidence interval
CJFH: specimen collection and processing by the China–Japan Friendship Hospital
CUADHS: Chinese Urban Adults Diet and Health Study
CV: coefficient of variation
CVD: cardiovascular disease
DBP: diastolic blood pressure
DRIs: Dietary Reference Intakes
EAR: estimated average requirements
FAO: Food and Agriculture Organisation of the United Nations
FFQ: food frequency questionnaire
FSG: fasting serum glucose
HDL: high-density lipoprotein
HFD: healthful food diversity
KNHANES: Korean National Health and Nutrition Examination Survey
MET: metabolic equivalents
MetS: metabolic syndrome
MPA: mean probability of adequacy
NHANES: National Health and Nutrition Examination Survey
PA: probabilities of nutrient adequacy
RNI: recommended nutrient intakes
SBP: systolic blood pressure
TC: total cholesterol
TG: triglyceride
WC: waist circumference
