# Supplementary material for: Using an introduced index to assess the association between food diversity and metabolic syndrome and its components in Chinese adults
Source: BMC Cardiovasc Disord. 2018 Oct 3;18:189. doi: 10.1186/s12872-018-0926-x (PMC6171175; doi:10.1186/s12872-018-0926-x)
Supplement: Supplementary file 1 — Table S1. Comparison of energy and macronutrients calculated from mean value of three-day 24-h dietary recalls and one-time 24-h dietary recall in the CUADHS. (DOCX 14 kb) [file 12872_2018_926_MOESM1_ESM.docx]

**Supplemental Table 1. Comparison of energy and macronutrients calculated from mean value of 3-days’ 24-hour dietary recalls and one time 24-hour dietary recall in CUADHS**

| Item | Number of subjects | Mean value of 3-days’ | One time 24-hour dietary recall | *P* |
| --- | --- | --- | --- | --- |
|  |  | Median(P25, P75) | |  |
| Energy, KJ | 205 | 6072.09(4800.98,8085.46) | 6614.96(5129.76,8358.98) | 0.151 |
| Preotein, g | 205 | 47.42(35.79,62.48) | 48.68(35.39,64.63) | 0.474 |
| Fat, g | 205 | 49.82(31.11,65.01) | 53.37(38.69,71.88) | 0.052 |
| Carbohydrates, g | 205 | 212.18(157.13,278.15) | 219.8(154.77,301.65) | 0.789 |
