# Supplementary material for: Using an introduced index to assess the association between food diversity and metabolic syndrome and its components in Chinese adults
Source: BMC Cardiovasc Disord. 2018 Oct 3;18:189. doi: 10.1186/s12872-018-0926-x (PMC6171175; doi:10.1186/s12872-018-0926-x)
Supplement: Supplementary file 2 — Table S2. Measurement methods of anthropometry indicators and blood biochemical indices. (DOCX 15 kb) [file 12872_2018_926_MOESM2_ESM.docx]

**Supplemental Table 2. Comparison of measurement methods of indices in two studies**

| Index | CHNS | CUADHS |
| --- | --- | --- |
| Waist circumference | Measured with an inelastic tape to the nearest 0.1 centimeter directly above the umbilicus for 1 centimeter around the waist at the end of exhalation , and do keep the tape level | Measured with an inelastic tape to the nearest 0.1 cm at a midpoint between the bottom of the rib cage and the top of the iliac crest at the end of exhalation |
| Triglycerides, TC | Measured by glycerol lipase oxidase(GPO-PAP) method in serum, which was collected by centrifuging the sample 3000g for 15minutes | Measured by enzymatic process (dissociation) method in serum, which was collected by centrifuging the sample 3000g for 15minutes |
| High-density lipoprotein cholesterol, HDL-C | Measured by enzymatic process(direct) method in serum, which was collected by centrifuging the sample 3000g for 15minutes | Same as CUADHS |
| Fasting glucose | Measured by glucose oxidase method(GOD) in serum, which was collected by centrifuging the sample 3000g for 15minutes | Same as CUADHS |
| Blood pressure | Measured by electronic sphygmomanometer (OMRON HEM-7124, Omron health (China) co. LTD) after the subjects 5 minutes of seated rest and no physical activity in 30 minutes for once, repeated once when the measurements were suspicious | Measured by standard mercury sphygmomanometers after 10 minutes of seated rest and a 30 s interval between cuff inflations for three times |
